# Increased epithelial stem cell traits in advanced endometrial endometrioid carcinoma

**DOI:** 10.1186/1471-2164-10-613

**Published:** 2009-12-16

**Authors:** Shing-Jyh Chang, Tao-Yeuan Wang, Chan-Yen Tsai, Tzu-Fang Hu, Margaret Dah-Tsyr Chang, Hsei-Wei Wang

**Affiliations:** 1 Institute of Microbiology and Immunology, National Yang-Ming University, Taipei, Taiwan; 2 VGH-YM Genome Research Center, National Yang-Ming University, Taipei, Taiwan; 3 Department of Obstetrics and Gynecology, Hsinchu Mackay Memorial Hospital, Hsinchu, Taiwan; 4 Institute of Molecular and Cellular Biology, National Tsing Hua University, Hsinchu, Taiwan; 5 Department of Pathology, Mackay Memorial Hospital, Taipei, Taiwan; 6 Mackay Medicine, Nursing and Management College, Taipei, Taiwan; 7 Department of Education and Research, Taipei City Hospital, Taipei, Taiwan

## Abstract

**Background:**

It has been recognized cancer cells acquire characters reminiscent of those of normal stem cells, and the degree of stem cell gene expression correlates with patient prognosis. Lgr5(+) or CD133(+) epithelial stem cells (EpiSCs) have recently been identified and these cells are susceptible to neoplastic transformation. It is unclear, however, whether genes enriched in EpiSCs also contribute in tumor malignancy. Endometrial endometrioid carcinoma (EEC) is a dominant type of the endometrial cancers and is still among the most common female cancers. Clinically endometrial carcinoma is classified into 4 FIGO stages by the degree of tumor invasion and metastasis, and the survival rate is low in patients with higher stages of tumors. Identifying genes shared between advanced tumors and stem cells will not only unmask the mechanisms of tumor malignancy but also provide novel therapeutic targets.

**Results:**

To identify EpiSC genes in late (stages III-IV) EECs, a molecular signature distinguishing early (stages I-II) and late EECs was first identified to delineate late EECs at the genomics level. ERBB2 and CCR1 were genes activated in late EECs, while APBA2 (MINT2) and CDK inhibitor p16 tumor suppressors in early EECs. MAPK pathway was significantly up in late EECs, indicating drugs targeting this canonical pathway might be useful for treating advanced EECs. A six-gene mini-signature was further identified to differentiate early from advanced EECs in both the training and testing datasets. Advanced, invasive EECs possessed a clear EpiSC gene expression pattern, explaining partly why these tumors are more malignant.

**Conclusions:**

Our work provides new insights into the pathogenesis of EECs and reveals a previously unknown link between adult stem cells and the histopathological traits of EECs. Shared EpiSC genes in late EECs may contribute to the stem cell-like phenotypes shown by advanced tumors and hold the potential of being candidate therapeutic targets and novel prognosis biomarkers.

## Background

Tumor development, progression, and prognosis remain at the front position of medical research. Two hypotheses of the origin of cancer have existed for many decades. One hypothesis postulates that adult stem or precursor cell is the cell of origin for cancer, whereas the other declares a somatic cell can be mutated and then be dedifferentiated or be reprogrammed to regain properties associated with both cancer cells and stem cells [1-3]. The discovery of a subpopulation of tumor stem cells (TSCs) in leukemia and solid cancers has strengthened the stem cell hypothesis [4]. Glioblastomas also possess characters and gene expression patterns of local neural stem cells (NSCs) [5], and artificially introducing cancer-associated mutations into stem or lineage-restricted precursor cells can indeed turn them into cancer initiating cells and all mice received mutations developed medulloblastomas [6,7]. Another example that the adult stem cell represents the cell of origin of cancer has recently been made in chronic myeloid leukemia (CML): by restricting BCR-ABLp210 expression to mouse Sca1(+) hematopoietic stem cells, it is sufficient to induce CML formation that recapitulates the human disease [8]. These evidences support the idea that mutations of stem cells may initiate the carcinogenic process of certain, although not necessary all, tumors.

On the other hand, the importance of somatic or tumor cell mutation and dedifferentiation has not been excluded completely. It has been recognized that during malignant transformation, cancer cells acquire genetic mutations that override the normal mechanisms controlling cellular proliferation. Human tumor cells can be created from healthy somatic cells with defined genetic elements [9]. Even though cancers were originated from mutated stem cells, newly acquired mutations in tumors still contribute in cell malignancy and therapy resistance. It has been recognized that cancer cells acquire characters reminiscent of those of normal stem cells. Clinically cancer cells with poor differentiated pathological grading usually have worse therapy response than those with well differentiated morphology. The degree of embryonic gene re-expression correlates with pivotal tumor features and patient prognosis [10,11]. It is known that colon cancers adopt a broad program encompassing embryonic colon development [12]. In poorly differentiated breast cancer, gliomas and bladder carcinoma, an embryonic stem cell (ESC)-like gene expression signature is exhibited and the degree of ESC program recapitulation correlates with tumor stages and patient survival [13]. Recent studies demonstrated that Snail, a potent oncogene which can induce epithelial-mesenchymal transition (EMT), contributes to the acquisition of stem cell traits in breast cancer cells [14,15]. Pre-existing cancerous lesions may become more malignant by the accumulation of new oncogenic mutations (such as Snail) that can induce cell dedifferentiation. Identifying genes shared between transformed cells, especially the more malignant ones, and stem cells will help to unmask the pathogenesis of tumors, as well as provide us with novel therapeutic targets and prognosis biomarkers.

Endometrial carcinoma of the female genital tract can be divided into two forms: endometrial endometrioid carcinoma (EEC; Type I) which account for 70-80% of cases and are estrogen-related; whereas the Type II tumors (papillary serous or clear cell tumors) account for 20% of cases unrelated to estrogen stimulation [16]. Clinically endometrial carcinoma is classified into 4 FIGO stages by the degree of invasion and metastasis: stage I tumors are limit to the uterine body and stage II tumors extend to the uterine cervix. Both stages are considered as less invasive, although stage IIB cases are characterized by a less favorable prognosis. In contrast, tumors of stages III-IV are invasive: for stage III there is regional tumor spread and for stage IV there is bulky pelvic disease or distant spread [17]. Approximately 72% of endometrial carcinomas are stage I, 12% are stage II, 13% are stage III, and 3% are stage IV [17]. The survival rate is also low in patients with higher stages of tumors: 80-90% in stage I, 70-80% in stage II, 40-60% in stage III, and 20% in stage IV [17]. Identifying genes abundant in late EECs can not only unmask the mechanisms of tumor malignancy but also provide us with novel therapeutic targets. Recently Lgr5- or CD133-positive crypt stem cells of the intestinal track were identified and these cells were proven to be one of the original cells of intestinal cancer [18,19]. OLFM4 is also a new, robust marker for stem cells in human intestine and marks a subset of colorectal cancer cells [20]. Disruption of beta-catenin in cells positive for CD133 resulted in a gross disruption of crypt architecture and a disproportionate expansion of CD133(+) cells at the crypt base [19]. It is unclear, however, whether genes high expressed in epithelial stem cells (EpiSCs) also contribute in tumor invasiveness, malignancy and therapy resistance. A broad description of stem cell traits reminiscent in EECs is therefore crucial.

In this study we dealt with the molecular bases of endometrial cancer and assessed the expression of epithelial precursor genes in advanced EEC. To examine the shared genes between EpiSC and late EECs, we first need to unmask the gene compositions in different stages of EECs. For this purpose we applied gene expression microarray and machine learning algorithms to filtrate genes differentially expressed in early (stages I-II) and late (stages III-IV) EECs. After obtaining genes unique in EECs of different stages, we then related transcriptional programs in EpiSCs and late EECs. This approach helped to discover a total of 217 probe sets differentiating EECs of different stages, and, moreover, showed late EECs possess a clear EpiSC gene expression pattern, partly explaining why these tumors are more malignant and fatal.

## Results

### Molecular signatures of early and late stage EECs

**Figure 1:**
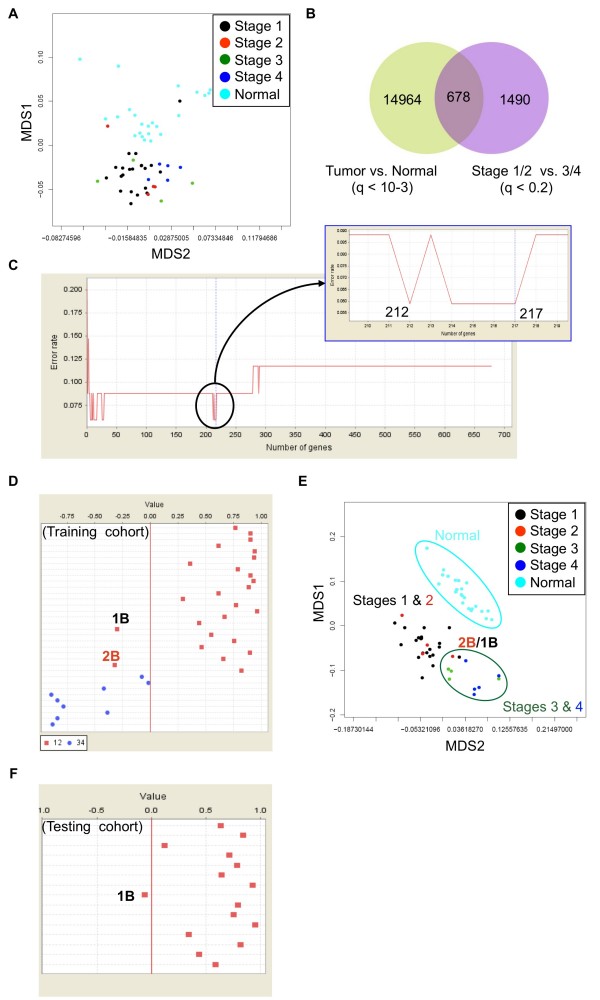
**Identification of genes in different EECs**. **(A) **A multidimensional scaling (MDS) plot drawn by all probe sets (~54600 ones) on the chip. Normal endometrium (Normal) and EECs of all 4 stages are included. Each spot represents an array. (**B**) A Venn diagram summarizing genes differentially expressed between normal and tumor tissues or between early (Stages 1 & 2) and late (Stages 3 & 4) EEC samples in the training cohort. (**C**) Narrowing down the existing gene signature using a machine learning strategy. When probe sets were ranked by signal-to-noise ratios (weights), the top 217 features was the largest panel to give the lowest error rate (i.e., a best classification effect; *upper panel*). (**D**) The discrimination ability of the 217-probeset signature. A prediction strength plot [25] shows the prediction strengths of the identified 217 probe sets in discriminating early from late EECs in the training cohort. Samples 1B and 2B denote 2 early EECs (Stages 1B and 2B, respectively) which express late EEC gene signatures. (**E**) A MDS plots using the above 217 probe sets. 2 misgrouped early EECs are indicated. (**F**) Signature evaluation by an independent testing data set. One Stage 1B case, which expresses late EEC gene signatures, is grouped into the late EEC area (separated by a red line).

To identify epithelial stem cell genes in late EECs, we first delineated early (FIGO stages I and II) and late (FIGO stages III and IV) EECs at the genomics level. We explored genes differentially expressed between early and late EEC tissues using the Affymetrix U133 Plus 2.0 array. The demographics of patients in the training and testing cohorts are in Tables 1 and 2, respectively. Tumor samples were compared to each other to minimize stromal and myometrial contamination as well as female-specific genes. A multidimensional scaling (MDS) plot using the whole transcriptome showed that the mRNA profiles of normal and cancerous tissues are different (Figure 1A). We then searched for genes distinguishing early and late EECs according to a statistical pipeline we used [21,22]. A total of 678 probe sets could differentiate early and late stage samples, as well as discriminate 23 normal endometrium and 33 tumor tissues (Figure 1B; the positive false discovery rate (pFDR) cutoff q values are shown).

**Table 1:** Characteristics of 34 EEC patients used in the training cohort.

GSE No.	TNM	FIGO stage	Histology	FIGO grade	Patient Age	Ethnic Background
***(Isolation site: Endometrium)***						
GSM117600	T1aN0M0	1A	Adenocarcinoma	1	60-70	Asian
GSM152644	T1bN0M0	1B	Endometrioid	2	60-70	Caucasian
GSM152660	T1bN0M0	1B	Endometrioid	2	40-50	Caucasian
GSM137960	T1bN0M0	1B	Endometrioid	2	60-70	Caucasian
GSM137968	T1bN0M0	1B	Endometrioid	2	60-70	Caucasian
GSM137980	T1bN0M0	1B	Endometrioid	3	40-50	Caucasian
GSM117586	T1bN0M0	1B	Endometrioid	2	50-60	African-American
GSM117643	T1bN0M0	1B	Endometrioid	1	70-80	Caucasian
GSM117667	T1bN0M0	1B	Endometrioid	2	60-70	Caucasian
GSM117703	T1bN0M0	1B	Endometrioid	2	50-60	Caucasian
GSM117704	T1bN0M0	1B	Endometrioid	2	50-60	Caucasian
GSM117722	T1bN0M0	1B	Endometrioid	2	70-80	Caucasian
GSM117724	T1bN0M0	1B	Endometrioid	2	60-70	Caucasian
GSM117739	T1bN0M0	1B	Endometrioid	3	60-70	Caucasian
GSM89034	T1bN0		Endometrioid	2	40-50	Caucasian
GSM89089	T1bN0M0	1B	Endometrioid	1	70-80	Caucasian
GSM76499	T1bN0M0	1B	Endometrioid	2	70-80	Caucasian
GSM76638	T1bN0M0	1B	Endometrioid	2	60-70	Caucasian
GSM117697	T1cN0M0	1C	Endometrioid	3	60-70	Caucasian
GSM89076	T1cN0M0	1C	Endometrioid	3	70-80	African Indian
GSM76507	T1cN0M0	1C	Endometrioid	2	60-70	Caucasian
GSM137955	T2aN0M0	2A	Endometrioid	2	60-70	African-American
GSM102425	T2bN0M0	2B	Endometrioid	2	50-60	Caucasian
GSM102444	T2bN0M0	2B	Endometrioid	1	60-70	Caucasian
GSM46912	T2bN0M0	2B	Endometrioid	1	60-70	Caucasian
						
GSM117708	T3aN0M0	3A	Endometrioid	3	70-80	Caucasian
GSM117712	T3aN0M0	3A	Endometrioid	2	60-70	Caucasian
GSM38067	T3aN0M0	3A	Endometrioid	3	60-70	Caucasian
GSM38084	T4NXM0	4A	Endometrioid	3	60-70	Caucasian
GSM89087	T3aNXM1 (*)	4B	Endometrioid	3	80-90	Caucasian
GSM46867	T3aN1M1 (**)	4B	Endometrioid	3	60-70	Caucasian
***(Isolation site: outside endometrium)***						
GSM89079	T3aNXM1($)	4B	Endometrioid	3	40-50	Caucasian
GSM203686	T3aN0M0 ($)	3A	Endometrioid	2	60-70	Caucasian
GSM46932	TXNXM1(@)	4B	Endometrioid	2	50-60	Caucasian

Endometrioid: Endometrioid carcinoma

*: Hepatic metastasis

**: Lymph node metastasis

$: Isolated from ovary

@: Isolated from abdominal wall fascia

**Table 2:** Characteristics of another 15 early EEC patients used in the testing set.

GSE No.	TNM	FIGO stage	Histology	FIGO grade	Patient Age	Ethnic Background
GSM88952	T1aN0M0	1A	Endometrioid	2	50-60	Caucasian
GSM76487	T1bN0M0	1B	Endometrioid	1	30-40	Caucasian
GSM102469	T1bN0M0	1B	Endometrioid	1	60-70	Caucasian
GSM117579	T1bN0MX	1B	Endometrioid	1	80-90	African-American
GSM117589	T1bN0MX	1B	Endometrioid	1	80-90	Caucasian
GSM117767	T1bN0MX	1B	Endometrioid	2	60-70	Caucasian
GSM137961	T1bN0MX	1B	Endometrioid	2	60-70	Caucasian
GSM117590	T1cN0MX	1C	Endometrioid	2	70-80	Caucasian
GSM117729	T2aN0M0	2A	Endometrioid	1	50-60	Caucasian
GSM53176	T2aN0MX	2A	Endometrioid	1	60-70	Caucasian
GSM117582	T2aN0MX	2A	Endometrioid	2	40-50	Caucasian
GSM88966	T2aN1M0	2A	Endometrioid	2	80-90	Caucasian
GSM53174	T2bN0MX	2B	Adenosarcoma	2	60-70	Caucasian
GSM76525	T1aN0M0	1A	Endometrioid (Mix)	3	80-90	Caucasian
GSM76632	T1bN0M0	1B	Adenocarcinoma	2	50-60	Caucasian

Mix: Mixed endometrioid and serous adenocarcinoma

The discrimination ability of these 678 probe sets were evaluated by a supervised machine learning strategy, which combines the weighted voting algorithm and leave-one-out cross validation (LOOCV) [23-25]. An error rate of 12.1% (2 out of 24 early cancers and 2 out of 9 late samples; P < 0.001 by permutation test) was found (Figure 1C and Additional file 1). However, we found the top 217 features (ranked by the weighted value of each probe set [25]) is the largest panel to have better discrimination ability than that of the 678-probeset signature (error rate 6.1% vs. 12.1%; Figure 1C, upper panel): 2 out of 24 early EEC tissues are classified into the late group while all 9 late ones are correct (Figure 1D). MDS analysis supports the superior classification power of these 217 probe sets: only 2 early samples express late EECs gene signatures and are grouped together with the late cases (Figure 1E). When applying these 217 probe sets on another independent testing data set containing 15 early EEC cases, 1 out of 15 early tissues (error rate 6.7%; P < 0.001 by permutation test) is misgrouped (Figure 1F).

### In-depth exploration of EEC-related genes

**Figure 2:**
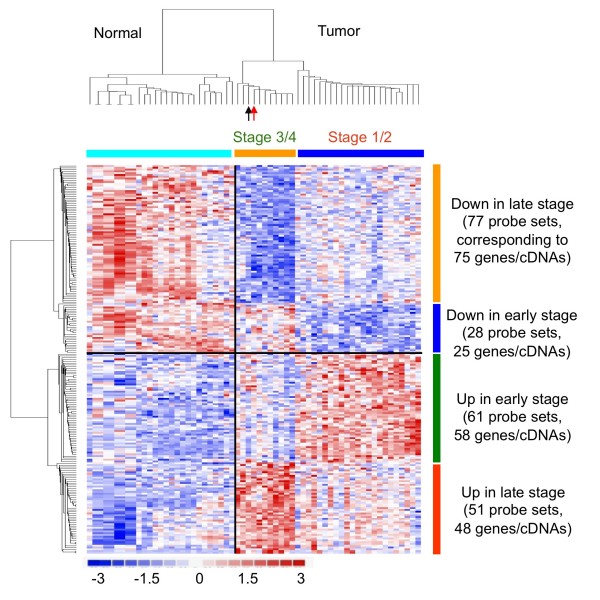
**Molecular fingerprint of EEC subtypes**. A heat map shows the 217 probes sets differentiating early and late EECs in the training data set, as well as discriminating normal endometrium and tumor tissues. Columns represent tumor samples; rows represent probe sets. In red, increased; in blue, decreased. Arrows indicates two early EECs which express a late EEC gene signature (black, Stage 1B; red, Stage 2B).

To have a better idea how the filtrated genes distribute in early and late EECs, a gene expression heat map for those 217 probe sets was drawn (Figure 2). This heat map showed the unique gene expression patterns between early or late EEC tumor tissues. Consistent with the classification data obtained by prediction strength (PS) analysis in Figure 1D, hierarchical clustering showed that only 2 early cases in the training data set are misclassified (indicated by arrows; Figure 2).

**Figure 3:**
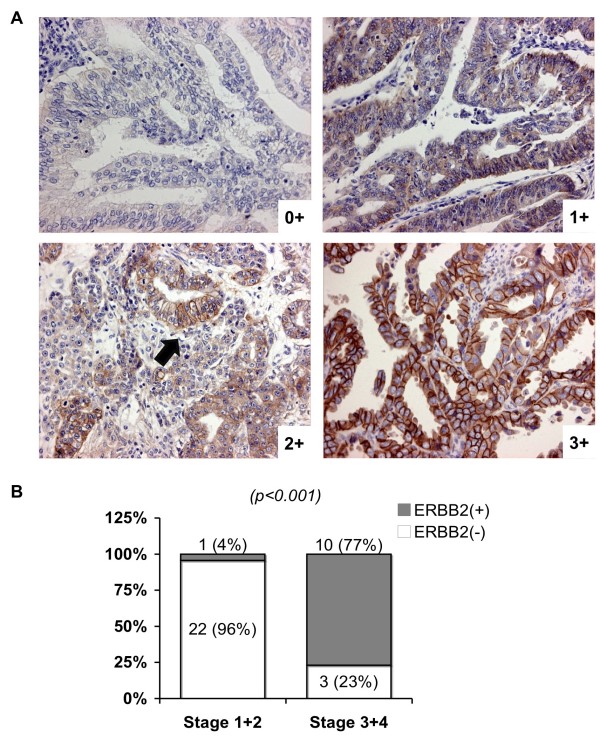
**ERBB2 protein expression in early and late EECs**. (**A**) Representative immunohistochemical (IHC) staining of ERBB2 protein in primary EEC tissues. Staining results were graded as 0+: undetectable staining in <10% of the tumor cells; 2+: weak to moderate complete membrane staining (indicated by an arrow) in <10% of the tumor cells; 3+: strong complete membrane staining observed in <10% of the tumor cells. EEC cases were categorized as ERBB2-negative (scores 0 and 1+) or positive (scores 2+ and 3+). (**B**) A histogram summarizing the IHC results on 36 primary EEC tissues stained for ERBB2. A chi square test P value is shown. Case numbers and percentages are also indicated.

Those 217 probe sets correspond to 177 known genes (with gene symbols) and 29 cDNAs, which have no gene symbols been assigned yet (all in Additional file 2). Among them 58 genes/cDNAs are predominantly up in early ECCs while 25 being down (Figure 2). In contrast, 48 genes/cDNAs are particularly high in late EECs while another 75 being low (Figure 2). The details of known genes (especially those with known function) are in Tables 3, 4, 5, 6 and 7 respectively. Many of these genes, such as CD163 [26], MSR1 (CD204) [27], ERBB2 oncogene (also known as HER-2/neu) [28,29], CSTA (stefin A) [30] and CCR1 [31], have been associated with tumor malignancy and poor patient outcomes in EEC or other cancers (Table 3, bold). CD163 and MSR1 (macrophage scavenger receptor 1; CD204) are markers for M2 macrophages, whose infiltration in tumor lesions is correlated with the histological grade of the gliomas [27] (Table 3, bold). These consistent findings support the reliability of our gene lists. We also validated our array data by performing immunohistochemical staining on Taiwanese EEC cases. ERBB2 was indeed more abundant in stages III and IV EEC tissues (Figure 3).

**Table 3:** Up-regulated known genes in late stage EECs.

Probe Set ID	UniGene ID	Gene Title	Gene Symbol	Chromosomal Location
213532_at	Hs.404914	ADAM metallopeptidase domain 17	ADAM17	chr2p25
223660_at	Hs.281342	adenosine A3 receptor	ADORA3	chr1p13.2
200966_x_at	Hs.513490	aldolase A, fructose-bisphosphate	ALDOA	chr16q22-q24
205568_at	Hs.104624	aquaporin 9	AQP9	chr15q22.1-22.2
224376_s_at	Hs.584985	chromosome 20 open reading frame 24	C20orf24	chr20q11.23
224972_at	Hs.472564	chromosome 20 open reading frame 52	C20orf52	chr20q11.22
200625_s_at	Hs.370581	CAP, adenylate cyclase-associated protein 1	CAP1	chr1p34.2
201850_at	Hs.516155	capping protein (actin filament), gelsolin-like	CAPG	chr2p11.2
205098_at	Hs.301921	chemokine (C-C motif) receptor 1	**CCR1**	chr3p21
203645_s_at	Hs.504641	CD163 molecule	**CD163**	chr12p13.3
209396_s_at	Hs.382202	chitinase 3-like 1 (cartilage glycoprotein-39)	CHI3L1	chr1q32.1
204971_at	Hs.518198	cystatin A (stefin A)	**CSTA**	chr3q21
202190_at	Hs.172865	cleavage stimulation factor, subunit 1, 50kDa	CSTF1	chr20q13.31
1554863_s_at	Hs.473133	docking protein 5	DOK5	chr20q13.2
224336_s_at	Hs.536535	dual specificity phosphatase 16	DUSP16	chr12p13
218282_at	Hs.632276	ER degradation enhancer mannosidase a-like 2	EDEM2	chr20q11.22
216836_s_at	Hs.446352	v-erb-b2 erythroblastic leukemia viral oncogene	**ERBB2 (HER2)**	chr17q11.2-q12
203561_at	Hs.78864	IgG Fc fragment, IIa, receptor (CD32)	FCGR2A	chr1q23
210889_s_at	Hs.352642	IgG Fc fragment, IIb, receptor (CD32)	FCGR2B	chr1q23
210992_x_at	Hs.78864	IgG Fc fragment, IIc, receptor (CD32)	FCGR2C	chr1q23.3
204007_at	Hs.176663	IgG Fc fragment, IIIb, receptor (CD16b)	FCGR3B	chr1q23
217782_s_at	Hs.268530	G protein pathway suppressor 1	GPS1	chr17q25.3
212355_at	Hs.558466	KIAA0323	KIAA0323	chr14q12
203364_s_at	Hs.410092	KIAA0652	KIAA0652	chr11p11.2
230252_at	Hs.155538	lysophosphatidic acid receptor 5	LPAR5	chr12p13.31
228360_at	Hs.357567	hypothetical protein LOC130576	LOC130576	chr2q23.2
226710_at	Hs.105685	similar to RIKEN cDNA C030006K11 gene	MGC70857	chr8q24
224324_at	Hs.131072	maestro	MRO	chr18q21
226241_s_at	Hs.355935	mitochondrial ribosomal protein L52	MRPL52	chr14q11.2
214770_at	Hs.632045	macrophage scavenger receptor 1	**MSR1 (CD204)**	chr8p22
205460_at	Hs.156832	neuronal PAS domain protein 2	NPAS2	chr2q11.2
209222_s_at	Hs.473254	oxysterol binding protein-like 2	OSBPL2	chr20q13.3
210907_s_at	Hs.478150	programmed cell death 10	PDCD10	chr3q26.1
238693_at	Hs.529592	Polyhomeotic like 3 (Drosophila)	PHC3	chr3q26.2
203691_at	Hs.112341	peptidase inhibitor 3, skin-derived (SKALP)	PI3	chr20q12-q13
226577_at	Hs.593811	Presenilin 1 (Alzheimer diseas 3)	PSEN1	chr14q24.3
217811_at	Hs.369052	selenoprotein T	SELT	chr3q25.1
222523_at	Hs.401388	SUMO1/sentrin/SMT3 specific peptidase 2	SENP2	chr3q27.2
227518_at	Hs.585896	solute carrier family 35, member E1	SLC35E1	chr19p13.11
1552671_a_at	Hs.496057	solute carrier family 9 (Na/H exchanger), 7	SLC9A7	chrXp11.3-11.23
222410_s_at	Hs.583855	sorting nexin 6	SNX6	chr14q13.2
203114_at	Hs.25723	Sjogren's syndrome/scleroderma autoantigen 1	SSSCA1	chr11q13.1
223478_at	Hs.530373	translocase of inner mitochondrial 8 homolog B	TIMM8B	chr11q23.1-q23.2
212769_at	Hs.287362	transducin-like enhancer of split 3	TLE3	chr15q22
204787_at	Hs.8904	V-set and immunoglobulin domain containing 4	VSIG4	chrXq12-q13.3
221247_s_at	Hs.900069	Williams-Beuren syndrome region 16	WBSCR16	chr7q11.23
202939_at	Hs.591501	zinc metallopeptidase (STE24 homolog, yeast)	ZMPSTE24	chr1p34
219050_s_at	Hs.121025	zinc finger, HIT type 2	ZNHIT2	chr11q13

**Table 4:** Down-regulated known genes in late stage EECs.

Probe Set ID	UniGene ID	Gene Title	Gene Symbol	Chromosomal Location
211224_s_at	Hs.158316	ATP-binding cassette, sub-family B, 11	ABCB11	chr2q24
232948_at	Hs.444414	AF4/FMR2 family, member 3	AFF3	chr2q11.2-q12
207133_x_at	Hs.99691	alpha-kinase 1	ALPK1	chr4q25
1562271_x_at	Hs.508738	Rho guanine nucleotide exchange factor 7	ARHGEF7	chr13q34
243899_at	Hs.579108	ADP-ribosylation factor-like 17 pseudogene 1	ARL17P1	chr17q21.32
211076_x_at	Hs.143766	Atrophin 1	ATN1	chr12p13.31
214256_at	Hs.128041	ATPase, Class V, type 10A	ATP10A	chr15q11.2
237716_at	Hs.434253	Chromosome 9 open reading frame 3	C9orf3	chr9q22.32
233844_at	Hs.522805	CD99 molecule-like 2	CD99L2	chrXq28
243640_x_at	Hs.127411	CDC14 cell division cycle 14 homolog A	CDC14A	chr1p21
233630_at	Hs.472027	CDP-diacylglycerol synthase 2	CDS2	chr20p13
210701_at	Hs.461361	craniofacial development protein 1	CFDP1	chr16q22.2-q22.3
238863_x_at	Hs.130849	Component of oligomeric golgi complex 8	COG8	chr16q22.1
215377_at	Hs.501345	C-terminal binding protein 2	CTBP2	chr10q26.13
1561616_a_at	Hs.591570	dynein, axonemal, heavy polypeptide 6	DNAH6	chr2p11.2
1560042_at	Hs.591566	family with sequence similarity 82, A	FAM82A	chr2p22.2
243588_at	Hs.403917	FERM, RhoGEF & pleckstrin domain protein 1	FARP1	chr13q32.2
243876_at	Hs.189409	Formin binding protein 1	FNBP1	chr9q34
1560094_at	Hs.155090	Guanine nucleotide binding protein, β 5	GNB5	chr15q21.2
210855_at	Hs.467733	GREB1 protein	GREB1	chr2p25.1
1557289_s_at	Hs.334930	GTF2I repeat domain containing 2	GTF2IRD2	chr7q11.23
232889_at	Hs.620129	glucuronidase, beta pseudogene 1	GUSBP1	chr5q13.2
1555685_at	Hs.463511	Hexose-6-phosphate dehydrogenase	H6PD	chr1p36
240482_at	Hs.519632	Histone deacetylase 3	HDAC3	chr5q31
1559600_at	Hs.632767	Hypermethylated in cancer 2	HIC2	chr22q11.21
1557329_at	Hs.371350	Holocarboxylase synthetase	HLCS	chr21q22.1
1553111_a_at	Hs.534040	kelch repeat and BTB domain containing 6	KBTBD6	chr13q14.11
231875_at	Hs.374201	kinesin family member 21A	KIF21A	chr12q12
232814_x_at	Hs.20107	Kinesin 2	KNS2	chr14q32.3
242112_at	Hs.631954	LSM11, U7 small nuclear RNA associated	LSM11	chr5q33.3
232418_at	Hs.30824	leucine zipper transcription factor-like 1	LZTFL1	chr3p21.3
1560033_at	Hs.167531	Methylcrotonoyl-Coenzyme A carboxylase 2	MCCC2	chr5q12-q13
216783_at	Hs.187866	Neuroplastin	NPTN	chr15q22
217802_s_at	Hs.632458	nuclear casein kinase and CDK substrate 1	NUCKS1	chr1q32.1
232644_x_at	Hs.518750	OCIA domain containing 1	OCIAD1	chr4p11
233270_x_at	Hs.491148	Pericentriolar material 1	PCM1	chr8p22-p21.3
1558695_at	Hs.188614	Pleckstrin homology domain containing, A5	PLEKHA5	chr12p12
233458_at	Hs.460298	polymerase (RNA) III polypeptide E	POLR3E	chr16p12.1
1566541_at	Hs.580351	Protein kinase C, epsilon	PRKCE	chr2p21
235004_at	Hs.519904	RNA binding motif protein 24	RBM24	chr6p22.3
212044_s_at	Hs.523463	Ribosomal protein L27a	RPL27A	chr11p15
215599_at	Hs.535014	SMA4	SMA4	chr5q13
1556784_at	Hs.551967	Smith-Magenis syndrome region, candidate 7	SMCR7	chr17p11.2
217704_x_at	Hs.628886	Suppressor of zeste 12 homolog pseudogene	SUZ12P	chr17q11.2
215279_at	Hs.499209	Supervillin	SVIL	chr10p11.2
207365_x_at	Hs.435667	thyroid hormone receptor, beta	THRB	chr3p24.2
215428_at	Hs.510833	Tight junction protein 1 (zona occludens 1)	TJP1	chr15q13
225004_at	Hs.514211	transmembrane protein 101	TMEM101	chr17q21.31
242347_at	Hs.8752	Transmembrane protein 4	TMEM4	chr12q15
238079_at	Hs.576468	tropomyosin 3	TPM3	chr1q21.2
237513_at	Hs.98609	trypsin X3	TRY1	chr7q34
1557571_at	Hs.439381	Vacuolar protein sorting 13 homolog D	VPS13D	chr1p36.22
235551_at	Hs.248815	WD repeat domain 4	WDR4	chr21q22.3
1555259_at	Hs.444451	sterile alpha motif and leucine zipper kinase AZK	ZAK	chr2q24.2

**Table 5:** Up-regulated biological modules in late EECs.

Biological Process	%	P-Value	Genes
Regulation of catalytic activity	12.50%	0.0053	DUSP16, **CAP1**, **ADORA3**, **ERBB2**, GPS1, **PSEN1**
Immune system process	16.67%	0.01694	AQP9, FCGR2A, FCGR2B, FCGR2C, FCGR3B, CCR1, ERBB2, VSIG4
Second-messenger-mediated signalling	8.33%	0.02006	**CAP1**, **ADORA3**, ERBB2, **CCR1**
Regulation of MAP kinase activity	6.25%	0.02205	**DUSP16**, ERBB2, **GPS1**
Cell surface receptor linked signal transduction	20.83%	0.02535	TLE3, CAP1, SENP2, ADORA3, ERBB2, LPAR5, CCR1, ADAM17, PSEN1, SNX6
Membrane organization and biogenesis	8.33%	0.0314	CAP1, ZMPSTE24, MSR1, TIMM8B

**Table 6:** Up-regulated known genes in early stage EECs.

Probe Set ID	UniGene ID	Gene Title	Gene Symbol	Chromosomal Location
225054_x_at	Hs.293560	Archaemetzincins-2	AMZ2	chr17q24.2
209870_s_at	Hs.525718	amyloid beta (A4) precursor protein-binding A2	**APBA2**	chr15q11-q12
1560851_at	Hs.351856	chromosome 10 open reading frame 136	C10orf136	chr10q11.21
234457_at	Hs.512758	chromosome 6 open reading frame 12	C6orf12	chr6p21.33
1561271_at	Hs.328147	coiled-coil domain containing 144C	CCDC144C	chr17p11.2
211156_at	Hs.512599	cyclin-dependent kinase inhibitor 2A (p16)	**CDKN2A**	chr9p21
220335_x_at	Hs.268700	esterase 31	CES3	chr16q22.1
204373_s_at	Hs.557659	centrosomal protein 350kDa	CEP350	chr1p36.13-q41
233502_at	Hs.12723	Contactin 3 (plasmacytoma associated)	CNTN3	chr3p26
244187_at	Hs.512181	Chromosome X open reading frame 33	CXorf33	chrXq21.1
229738_at	Hs.577398	dynein, axonemal, heavy polypeptide 10	DNAH10	chr12q24.31
219651_at	Hs.317659	developmental pluripotency associated 4	**DPPA4**	chr3q13.13
1555118_at	Hs.441145	ectonucleoside tri-P diphosphohydrolase 3	ENTPD3	chr3p21.3
206794_at	Hs.390729	v-erb-a erythroblastic leukemia viral oncogene	ERBB4	chr2q33.3-q34
241252_at	Hs.99480	establishment of cohesion 1 homolog 2	**ESCO2**	chr8p21.1
209631_s_at	Hs.406094	G protein-coupled receptor 37	**GPR37**	chr7q31
229714_at	Hs.171001	heparan sulfate 6-O-sulfotransferase 3	HS6ST3	chr13q32.1
213598_at	Hs.533222	Dimethyladenosine transferase	HSA9761	chr5q11-q14
231500_s_at	Hs.444600	SLC7A5 pseudogene	LAT1-3TM	chr16p11.2
232953_at	Hs.566209	hypothetical LOC400723	LOC400723	chr11p15.5
239076_at	Hs.520804	Similar to cell division cycle 10 homolog	LOC441220	chr7p13
1558579_at	Hs.587089	hypothetical protein LOC642691	LOC642691	chr2p11.1
222159_at	Hs.497626	Plexin A2	PLXNA2	chr1q32.2
226766_at	Hs.13305	roundabout, axon guidance receptor, 2	**ROBO2**	chr3p12.3
1569124_at	Hs.267765	similar to Leucine-rich repeat protein SHOC-2	RP11-139H14.4	chr13q14.12
220232_at	Hs.379191	stearoyl-CoA desaturase 5	SCD5	chr4q21.22
214257_s_at	Hs.534212	SEC22 vesicle trafficking protein homolog B	SEC22B	chr1q21.1
242536_at	Hs.205816	Solute carrier family 17, member 1	SLC17A1	chr6p23-p21.3
220551_at	Hs.242821	solute carrier family 17, member 6	SLC17A6	chr11p14.3
1559208_at	Hs.437696	ST7 overlapping transcript 4 (non-coding RNA)	ST7OT4	chr7q31.1-7q31.2
233251_at	Hs.21379	Spermatid perinuclear RNA binding protein	STRBP	chr9q33.3
223751_x_at	Hs.120551	toll-like receptor 10	TLR10	chr4p14
217797_at	Hs.301412	ubiquitin-fold modifier conjugating enzyme 1	UFC1	chr1q23.3
229997_at	Hs.515130	vang-like 1 (van gogh, Drosophila)	**VANGL1**	chr1p11-p13.1
204590_x_at	Hs.592009	vacuolar protein sorting 33 homolog A	VPS33A	chr12q24.31
232964_at	Hs.488157	Williams Beuren syndrome region 19	WBSCR19	chr7p13
227621_at	Hs.446091	Wilms tumor 1 associated protein	WTAP	chr6q25-q27
240296_at	Hs.98322	Zinc finger, A20 domain containing 1	ZA20D1	chr1q21.2
226208_at	Hs.593643	zinc finger, SWIM-type containing 6	ZSWIM6	chr5q12.1

**Table 7:** Down-regulated known genes in early stage EECs.

Probe Set ID	UniGene ID	Gene Title	Gene Symbol	Chromosomal Location
215535_s_at	Hs.409230	1-acylglycerol-3-phosphate O-acyltransferase 1	AGPAT1	chr6p21.3
202204_s_at	Hs.295137	autocrine motility factor receptor	AMFR	chr16q21
212536_at	Hs.478429	ATPase, Class VI, type 11B	ATP11B	chr3q27
220975_s_at	Hs.201398	C1q and tumor necrosis factor related protein 1	C1QTNF1	chr17q25.3
224794_s_at	Hs.495230	cerebral endothelial cell adhesion molecule 1	CEECAM1	chr9q34.11
1557394_at	Hs.249600	discs, large homolog-associated protein 4	DLGAP4	chr20q11.23
211958_at	Hs.369982	insulin-like growth factor binding protein 5	IGFBP5	chr2q33-q36
225303_at	Hs.609291	kin of IRRE like (Drosophila)	KIRREL	chr1q21-q25
218717_s_at	Hs.374191	leprecan-like 1	LEPREL1	chr3q28
209205_s_at	Hs.436792	LIM domain only 4	LMO4	chr1p22.3
203506_s_at	Hs.409226	mediator of RNA polymerase II transcription 12	MED12	chrXq13
207564_x_at	Hs.405410	O-linked N-acetylglucosamine transferase	OGT	chrXq13
214484_s_at	Hs.522087	opioid receptor, sigma 1	OPRS1	chr9p13.3
203244_at	Hs.567327	peroxisomal biogenesis factor 5	PEX5	chr12p13.3
241916_at	Hs.130759	Phospholipid scramblase 1	PLSCR1	chr3q23
229001_at	Hs.601513	Protein phosphatase 1, regulatory 3E	PPP1R3E	chr14q11.2
208720_s_at	Hs.282901	RNA binding motif protein 39	RBM39	chr20q11.22
209148_at	Hs.388034	retinoid × receptor, beta	RXRB	chr6p21.3
209352_s_at	Hs.13999	SIN3 homolog B, transcription regulator (yeast)	SIN3B	chr19p13.11
221500_s_at	Hs.307913	syntaxin 16	STX16	chr20q13.32
220036_s_at	Hs.272838	syntaxin 6	STX6	chr1q25.3
201110_s_at	Hs.164226	thrombospondin 1	THBS1	chr15q15
221507_at	Hs.631637	transportin 2 (importin 3, karyopherin b 2b)	TNPO2	chr19p13.13
208723_at	Hs.171501	ubiquitin specific peptidase 11	USP11	chrXp11.23

To gain more insights into the functional consequences of differential gene expression, we performed gene set enrichment analysis for the filtrated genes. Signature probe sets were subjected into the Gene Ontology (GO) database search to find statistically over-represented functional groups within these genes. The biological processes being statistically overrepresented (P < 0.05) in late stage-enriched genes are shown in Table 5. These predominant processes include those pertaining to immune system process, second-messenger-mediated signaling (genes also involved in cyclic nucleotide second messenger (P = 0.0306) are bold), MAP kinase activity (genes also involved in the inactivation of MAPK activity (P = 0.0459) are bold), membrane organization and biogenesis, regulation of catalytic activity (genes also involved in the positive regulation of catalytic activity (P = 0.0182) are bold), and cell surface receptor-linked signal transduction are significantly up (Table 5).

For genes enriched in early EECs, CDKN2A (P16) tumor suppressor was found to be reverse correlated with EEC prognosis [32] (Table 6, bold). Another tumor suppressor is APBA2 (amyloid beta (A4) precursor protein-binding, family A, member 2; also known as MINT2), which is frequently methylated and silent in colorectal carcinoma and gastric carcinoma [33]. Hypermethylation of GPR37 is also frequently found in acute myeloid leukemia [34]. In terms of oncogenes, ROBO2 (roundabout, axon guidance receptor, 2), a receptor of the SLIT2 axon guidance and cell migration growth factor, is associated with poor prognosis of breast cancer [35]. ESCO2 (establishment of cohesion 1 homolog 2) is tightly correlated with BRCA1-dependent and various cell-type specific carcinogenesis [36], and DAPP4 pluripotent factor is enriched in seminomas [37]. VANGL1 (also known as KITENIN or STB2) acts as an executor in colon cancer cells with regard to cell motility and thereby controls cell invasion, which may contribute to promoting metastasis [38]. The abundant expression of known oncogenes in early EECs also suggests the early EEC cases contain high percentage of epithelial tumor cells instead of merely stromal and myometrial contaminations.

### A six-gene signature distinguishing early and late EECs

**Figure 4:**
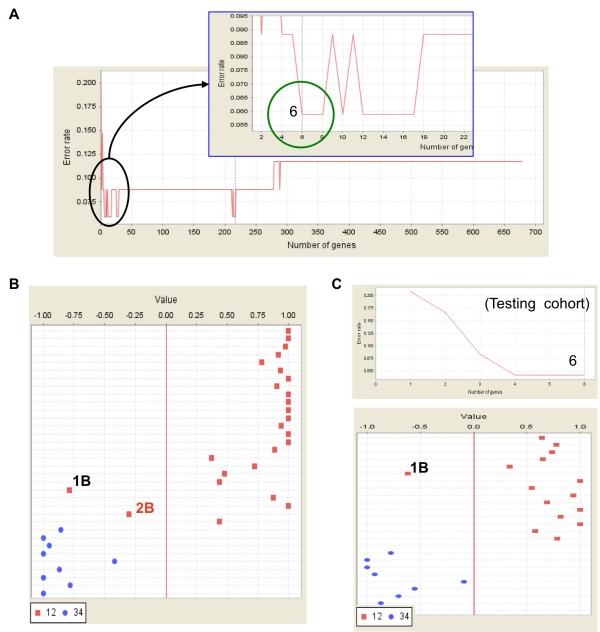
**A six-gene signature dividing early and late EECs**. (**A**) Further narrowing down the existing gene signature to fewer genes. When probe sets were ranked by their signal-to-noise ratios (weights), the top 6 features form the smallest panel which can give the best classification effect. (**B**) A prediction strength (PS) plot shows the prediction strength of these 6 genes. They give the same classification effect as that of the 217-probeset signature. (**C**) Signature evaluation by a testing data set. A lowest error rate (*upper*) and best classification effect (shown by a PS plot; *lower panel*) was achieved.

When evaluating the classification effect of filtrated genes, we noticed that the top 6 genes could already distinguish early and late EECs, and these 6 genes gave the same diagnostic power to that of the 217 probe sets in the training cohort (Figure 4A). The same two early cases (one Stage 1B and one Stage 2B) were misgrouped with the late ones (Figure 4B). When applying these 6 genes on the testing data set, a lowest error rate could also be achieved (Figure 4C, upper panel). Only 1 out of 15 early tissues (error rate 6.7%; P < 0.001 by permutation test) was misgrouped (Figure 4C, lower panel). The same Stage 1B sample was misclassified when either applied only these 6 genes or the entire 217 probe sets (Figure 1F). Thus, these 6 genes hold clinical potentials of being diagnostic biomarkers. These 6 genes are: (1) ATP-binding cassette, B (MDR/TAP), 11 (ABCB11) (2) Archaemetzincins-2 (AMZ2) (3) amyloid beta (A4) precursor protein-binding A2 (APBA2) (4) LIM domain only 4 (LMO4) (5) Hypothetical protein LOC647065 (LOC647065) and (6) Homo sapiens mRNA, clone IMAGE:5759975 (cDNA FLJ12258 fis) (Table 8). AMZ and APBA2 are up-regulated in early EECs. ABCB11, LOC647065 and cDNA FLJ12258 fis are down in tumors, especially in late EECs, while LMO4 particularly down in early EECs.

**Table 8:** Gene annotations of the six-gene signature.

Probe Set ID	UniGene ID	Gene Title	Gene Symbol	Chromosomal Location
233113_at	Hs.633901	Homo sapiens, clone IMAGE:5759975, mRNA	---	---
211224_s_at	Hs.158316	ATP-binding cassette, B (MDR/TAP), 11	ABCB11	chr2q24
225054_x_at	Hs.293560	Archaemetzincins-2	AMZ2	chr17q24.2
209870_s_at	Hs.525718	amyloid beta (A4) precursor protein-binding A2	APBA2	chr15q11-q12
209205_s_at	Hs.436792	LIM domain only 4	LMO4	chr1p22.3
239819_at	Hs.624027	Hypothetical protein LOC647065	LOC647065	chr2q23.1

### Re-activation of epithelial stem cell genes in advanced EECs

**Figure 5:**
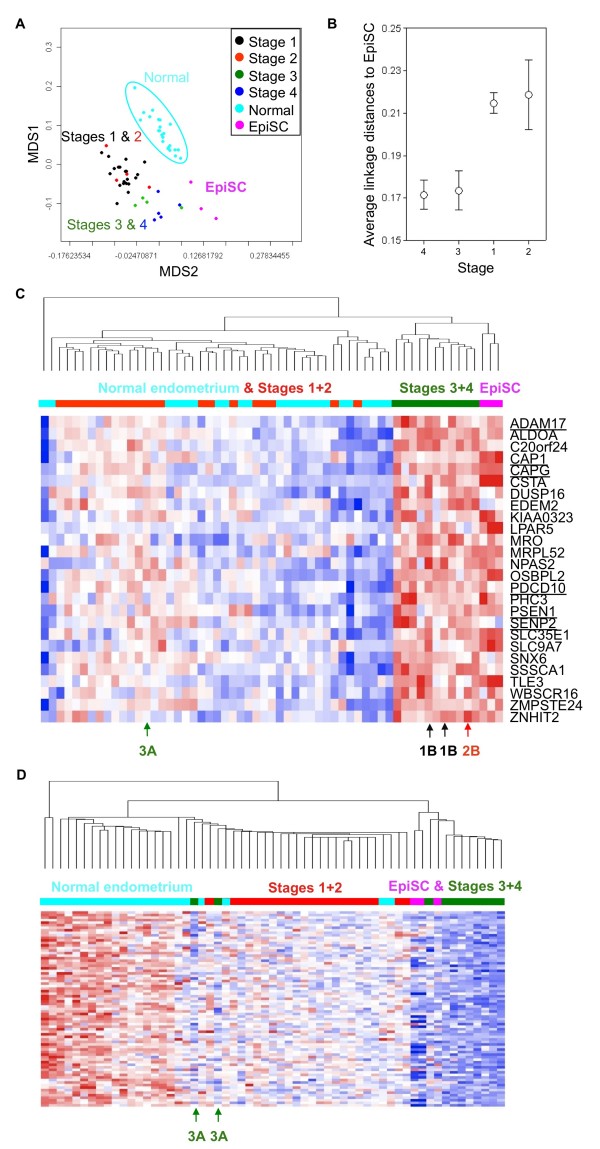
**Expression of EpiSC gene patterns in EECs, especially late ones**. (**A**) Relationships between normal endometrium, EECs of different stages in the training data set and epithelial stem cells (EpiSCs). This MDS plot was drawn by the 217 features differentiating early and late EECs. (**B**) Average linkage distances between tissues and EpiSCs. The same 217 probe sets were used. The confidence limits shown represent the standard error. (**C**) A heat map shows genes overexpressed in both EpiSCs and late EECs. Gene symbols of these genes are shown. Genes associate with tumor malignancy or stem cell biology are underlined. (**D**) A heat map shows the distribution patterns of the 77 probe sets down-regulated in late EECs. These genes are also absence in EpiSCs.

Since our main goal is to identify EpiSC genes in EECs, we compared the gene expression profiles of EEC tissues of all 4 stages to that of normal CD133+ EpiSCs [39]. When the 217 genes distinguishing early and late EECs were applied to compare the relationships between EECs and EpiSCs, clearly EpiSCs have a closest relationship to late EECs (Figure 5A). This impression is strengthened by calculating the average linkage distances between sample groups. Compared with early EECs, EEC of both Stages III and IV are closer to EpiSCs to a similar extent (Figure 5B), suggesting the re-expression of EpiSC features in late EECs. A total of 26 EpiSC genes are overexpressed in advanced EECs (Figure 5C). Also, genes down-regulated in late EECs (the 77 probe sets in Figure 2) are absence in EpiSCs (Figure 5D). Most early EECs clustered together and expressed the intermediate level of EpiSC genes (Figure 5C-D), consistent with the distances analysis result in Figure 5B.

## Discussion

EEC still ranks one of the most fatal female cancers worldwide and disease progression very often accompany with worse clinical outcomes and treatment failure. Identifying genes or canonical pathways associated with advanced cancer can help to unmask the mechanisms of tumor malignancy as well as provide us with novel drug targets. It has been recognized clinically that cancer cells, especially the advanced and metastatic ones, possess characters reminiscent of those of normal stem cells. The degree of stem cell gene expression correlates with pivotal tumor features and patient prognosis [10,11,13]. Hence, identifying shared genes between late EECs and stem cells will provide new insights into cancer biology, as well as new prognosis markers and therapeutic targets. In this study, we identified a 217-probeset signature which could distinguish late (stages III-IV) from early (stages I-II) EECs (Figure 1). More low stage disease array data than high stage ones were obtained, which may partly due to the fact that the early diagnosis takes place in almost 90% of EEC clinically. We combined primary and metastatic late EEC samples in one group since their molecular profiles are indistinguishable (not shown). Prostate EpiSCs were used as a comparative group since array data for endometrial stem cells is not available yet. Nevertheless, prostate CD133+ cells are still epithelial stem cells and therefore good controls. Other EpiSC data should reproduce part of our findings.

Our results reveal a previously unaware link between genes associated with EpiSC identity and the histopathological traits of EECs. It is possible that these genes contribute to the stem cell-like phenotypes of late EECs. A total of 26 EpiSC genes were found overexpressed in late EECs (Figure 5C), and genes down-regulated in late EECs (Figure 2; 77 probe sets) are also absence in EpiSCs (Figure 5D). Among those 26 overexpressed genes there are famous oncogenes or stemness genes (Figure 5C, underlined). ADAM17 (A Disintegrin and A Metalloproteinase 17), also known as tumor necrosis factor-alpha converting enzyme (TACE) or less commonly CD156q, is a therapeutic target in multiple diseases since major contemporary pathologies like cancer, inflammatory and vascular diseases seem to be connected to its cleavage abilities [40]. CAP1 (adenylate cyclase-associated protein 1) overexpressed in pancreatic cancers is involved in cancer cell motility [41]. CAPG (capping protein (actin filament), gelsolin-like) also contributes in the motility of pancreatic cancer cells [42]. PDCD10 (CCM3) is involved in cerebral cavernous malformations (CCM) [43] and is found to interact with Ste20-related kinase MST4 to promote cell growth and transformation via modulation of the ERK pathway [44]. PSEN1 (presenilin 1) is involved in apoptosis, overexpressed in high-risk patients with stage I non-small cell lung cancer (NSCLC), and is in a prognosis signature of NSCLC patients [45]. SENP2 (SUMO-specific protease 2) is highly expressed in trophoblast cells that are required for placentation, and targeted disruption of SENP2 in mice reveals its essential role in development of all three trophoblast layers via modulating the Mdm2-p53 pathway [46]. The appearance of these known oncogenes or stemness genes in our data supports the reliability of our gene lists. The roles of EpiSC genes in both epithelial stem cell biology and EEC malignancy will be addressed further.

Several genes were previous suggested to be tumor suppressors. CSTA (cystatin A, or stefin A), a cysteine proteinases inhibitor, is implicated in preventing local and metastatic tumor spread of cancers. The risk of disease recurrence and disease-related death was thus higher in patients with low CSTA in patients with squamous cell carcinoma of the head and neck [30]. NPAS2 (neuronal PAS domain protein 2) is a circadian gene as well as a putative tumor suppressor involved in DNA damage response [47]. PHC3 (polyhomeotic homolog 3), a component of the hPRC-H complex, associates with E2F6 during G0 and is lost in osteosarcoma tumors [48]. Validating their expression in different stages of EECs by further immunohistochemstry study will not only provide novel malignancy mechanisms but will also present new drug targets.

In the past few years, much effort has been put to explore the mechanisms and additional molecular markers for predicting prognosis of EECs by using high-throughput genomics technology. Gene expression microarray (GEM) is a popular platform among all of those high-throughput genomics techniques. In this study we applied GEM and machine learning algorithms to filtrate out a 217-probeset signature for disease diagnosis. Many of the filtrated genes have been linked to tumor progression and malignancy, supporting the reliability of our array data. Moreover, we narrowed down this 217-probeset profile to a six-gene mini-signature for the differentiation of early to late EECs in the training set. This signature can be validated by an independent testing cohort (Figure 4). Owing to the small gene number of this signature, it is now possible to check their mRNA levels in patient tissues by real-time PCR in regular clinical labs. Recently a five-gene profile and a five-microRNA signature are identified for the prediction of clinical outcomes in non-small-cell lung cancer [49,50]. Whether our six-gene signature can be correlated with relapse-free and overall survival among patients with EEC is unclear and awaited to be elucidated. Also, whether the protein expression levels of these 6 genes correlate with those of mRNAs is unclear. Since most of the patients in either training or testing data set were Caucasian (Table 1), whether this gene signature can be applied in patients with various genetic backgrounds should also be studied.

In our datasets we noticed that few early EEC cases expressed already late EEC genes and therefore could not be classified correctly (Figs. 1, 2). Since patients with late and metastatic EEC tend to have poor prognosis, whether these unusual early cases possess worse clinical outcomes is an interesting issue. It has been suggested that prognosis potential of human tumors is inherited in early lesions. For example, the gene expression patterns in metastatic colorectal carcinoma are readily distinguishable from those associated with *in situ *tumors [24,51]. A subset of primary tumors resembled metastatic tumors with respect to this gene-expression signature [24,51]. Very recently Varmus and colleagues showed that when untransformed mouse mammary cells were introduced into the systemic circulation of a mouse, those cells can bypass transformation at the primary site, form long-term residence in the lungs but do not form ectopic tumors [52]. Husemann et al. also observed that systemic spread can be an early step in breast cancer. Tumor cells can disseminate systemically from earliest epithelial alterations and form and micrometastasis in bone marrow and lungs [53]. Therefore, release from dormancy of early-disseminated cancer cells may frequently account for metachronous metastasis. The metastatic potential of human tumors is encoded in the bulk of a primary tumor and, at least in a subset of patients, metastatic capability in cancers is an inherent feature. Our EEC gene signatures therefore hold the potential of being a novel prognosis panel. More advanced therapy and clinical follow-up should be applied on early stage patients with molecular feature similar to that of EpiSC.

In advanced EECs, tumor tissues express more genes abundant in CD133+ EpiSC and acquired a stem cell trait (Figure 5). The expression of these EpiSC genes in late EECs may due to the re-expression of EpiSC features in late stage EECs, i.e., further mutations and stem cell gene reactivation in certain early EECs. The intermediate EpiSC gene expression level in early EECs supports this point (Figure 5A &5C-D). Recent studies demonstrated that EMT contributes to the acquisition of stem cell traits in cancer cells and the induction of EMT inducer Snail results in stemness gene expression [14,15]. Whether EMT also contributes in EEC progression and metastasis is an interesting issue to follow. However, we did not rule out the possibility that certain late EECs may arise from an independent rapidly progressing cancer utilizing stemness molecular pathways. According to the tumor stem cell theory, cancer cells may be originated from different cancer stem cells acquiring distinctive oncogenic mutations. Certain early EECs have the capacity to progress to late stage disease may due to a mechanism that they arose from the same mutated progenitor cells as late EECs. The observation that several early EEC cases express EpiSC genes already (Figure 1D &5C) favors the later hypotheses. These 2 situations may both exist *in vivo*, but our profiling work cannot favor any of them yet. Nevertheless, genes filtrated here will provide clinicians novel prognosis markers and therapeutic targets.

## Conclusions

In summary, here we reveal distinct epithelial stem cell traits and gene expression patterns in late EECs and some of these genes hold the potential of being novel drug targets. Drugs targeting MAP kinase pathway, for example, may be applied for the treatment of late EEC since this canonical pathway is significantly up in late EECs (Table 5). Since applying a statistical analysis of gene ontology terms is the reliance on prior knowledge of the biological activity of each differentially expressed gene, the enrichment of genes associated with specific pathways may be a consequence of intense research in such areas. Hence, new canonical pathways may still exist and may serve as candidate therapeutic targets. Function of the filtrated KIAA (such as KIAA0323, Figure 5C) and LOC series of anonymous ESTs (such as C20orf24, Figure 5C) in Tables 3, 4, 5, 6, 7 should be studied and their roles in tumor malignancy, chemoresistance and EpiSC stemness are awaited to be elucidated. Further studies to prove the prognosis values and therapeutic potentials of the identified genes, especially those also present in epithelial stem cells, should lead to a better understanding of EEC and EpiSC biology and the susceptibilities of late EECs to treatment.

## Methods

### Microarray data sets

All array data were implemented by the Affymetrix™ HG-U133 Plus 2.0 GeneChip. Array data of normal CD133+ epithelial stem cells, which were used as a normal counterpart of cancer stem cells [39], isolated from benign prostatic hyperplasia were downloaded from the ArrayExpress database at the European Bioinformatics Institute (http://www.ebi.ac.uk/microarray-as/ae/; Accession No. E-MEXP-993; array data files 1325504978.cel, 1325505459.cel and 1325505089.cel were used).

The gene expression profiles of EEC tissues of different stages were generated by the International Genomics Consortium (IGC) under the expO (Expression Project for Ontology) project and were downloaded from Gene Expression Omnibus (GEO http://www.ncbi.nlm.nih.gov/geo/; GSE2109). EEC array data were divided into training (n = 33; incl. all 4 stages) and testing cohorts (n = 15) (details in Table 1). Array data of normal endometrium controls were from a Human body index dataset in GEO (GSE7307).

### Array data processing

Feature selection was performed as previously described [22]. Briefly, the default robust multichip average (RMA) settings were used to background correct, normalize and summarize all expression values using the 'affy' package of the Bioconductor suite of software http://www.bioconductor.org/ for the R statistical programming language. A t-statistic was calculated as normal for each gene and a *p*-value then calculated using a modified permutation test in the "LIMMA" package [22]. To control the multiple testing errors, a false discovery rate (FDR) algorithm was then applied to these *p*-values to calculate a set of *q*-values: thresholds of the expected proportion of false positives, or false rejections of the null hypothesis [22,54]. Gene annotation was performed by the ArrayFusion web tool http://microarray.ym.edu.tw/tools/arrayfusion/[55]. Gene enrichment analysis was performed by the Gene Ontology (GO) database using the DAVID Bioinformatics Resources 2008 interface http://david.abcc.ncifcrf.gov/, a graph theory evidence-based method to agglomerate gene or protein identifiers [56,57].

### Bioinformatics analysis

The discrimination power of filtrated genes was evaluated by a machine-learning approach combining the weighted voting algorithm [24] and leave-one-out cross-validation (LOOCV). This approach has been integrated in our Java tool http://microarray.ym.edu.tw/tools/set/[25]. In brief, the uploaded genes are ranked according to the absolute values of corresponding signal-to-noise scores [24] in a descending order. Genes are included into a signature one at a time based on the order of ranking. The error rate for each new signature is estimated by the weighted voting algorithm and LOOCV and can be monitored by an error rate distribution plot [25]. Based on the error rate information, we then selected an appropriate composition of discriminating genes with the lowest error rate. Once a signature is defined, the result of prediction strength (PS) analysis for each sample was shown. The PS values range from -1 to +1, where higher absolute values reflect stronger predictions [25]. An overview of the results for samples in different groups was then illustrated by a PS plot [25].

Classical multidimensional scaling (MDS) is performed by the standard function of the R program to provide a visual impression of how the various sample groups are related. The average linkage distance between samples is calculated by the Pearson correlation subtracted from unity to provide bounded distances in the range (0, 2), as described in our previous study [22]. The distance between two groups of samples is calculated using the average linkage measure (the mean of all pair-wise distances (linkages) between members of the two groups concerned). The standard error of the average linkage distance between two groups (the standard deviation of pair-wise linkages divided by the square root of the number of linkages) is quoted when inter-group distances are compared in the text.

### Immunohistochemical staining

Staining was performed on formalin-fixed, paraffin-embedded specimens using anti-ERBB2 primary antibody (DAKO, Carpinteria, CA, USA). Scoring was performed as following. 0: undetectable staining or membrane staining in <10% of the tumor cells. 1+: faint and incomplete membrane staining in >10% of the tumor cells; 2+: weak to moderate complete membrane staining in >10% of the tumor cells; 3+: strong complete membrane staining observed in >10% of the tumor cells. ERBB2 protein expression was categorized as negative (scores 0 and 1+), or positive (scores 2+ and 3+) [29].

## Authors' contributions

SJC, TYW, and HWW designed the study project. SJC and TYW collected microarray data sets and EEC materials. SJC, TYW, CYT, and TFW executed project plan and data analysis. SJC, TYW, MDC, and HWW carried out data interpretation and discussion. SJC wrote the manuscript. Then HWW modified it. All authors read and approved the final manuscript.

## Supplementary Material

Additional file 1 **The discrimination ability of the 678 probe sets**. Prediction power of the 678 probe sets differentiating early and late stage samples, as well as discriminating normal endometrium and tumor tissues. Click here for file

Additional file 2 **The annotation of probed genes and cDNAs**. Complete data of analyzed arrays and clustered genes/cDNAs. Click here for file

## References

[B1] Yilmaz OH Valdez R Theisen BK Guo W Ferguson DO Wu H Morrison SJ Pten dependence distinguishes haematopoietic stem cells from leukaemia-initiating cells Nature 2006 441 7092 475 482 10.1038/nature04703 16598206

[B2] Barnhart BC Simon MC Metastasis and stem cell pathways Cancer Metastasis Rev 2007 26 2 261 271 10.1007/s10555-007-9053-3 17647111 PMC3215288

[B3] Trosko JE Review paper: cancer stem cells and cancer nonstem cells: from adult stem cells or from reprogramming of differentiated somatic cells Vet Pathol 2009 46 2 176 193 19261629 10.1354/vp.46-2-176

[B4] Reya T Morrison SJ Clarke MF Weissman IL Stem cells, cancer, and cancer stem cells Nature 2001 414 6859 105 111 10.1038/35102167 11689955

[B5] Lee J Kotliarova S Kotliarov Y Li A Su Q Donin NM Pastorino S Purow BW Christopher N Zhang W Park JK Fine HA Tumor stem cells derived from glioblastomas cultured in bFGF and EGF more closely mirror the phenotype and genotype of primary tumors than do serum-cultured cell lines Cancer Cell 2006 9 5 391 403 10.1016/j.ccr.2006.03.030 16697959

[B6] Yang ZJ Ellis T Markant SL Read TA Kessler JD Bourboulas M Schuller U Machold R Fishell G Rowitch DH Wainwright BJ Wechsler-Reya RJ Medulloblastoma can be initiated by deletion of Patched in lineage-restricted progenitors or stem cells Cancer Cell 2008 14 2 135 145 10.1016/j.ccr.2008.07.003 18691548 PMC2538687

[B7] Schuller U Heine VM Mao J Kho AT Dillon AK Han YG Huillard E Sun T Ligon AH Qian Y Ma Q Alvarez-Buylla A McMahon AP Rowitch DH Ligon KL Acquisition of granule neuron precursor identity is a critical determinant of progenitor cell competence to form Shh-induced medulloblastoma Cancer Cell 2008 14 2 123 134 10.1016/j.ccr.2008.07.005 18691547 PMC2597270

[B8] Perez-Caro M Cobaleda C Gonzalez-Herrero I Vicente-Duenas C Bermejo-Rodriguez C Sanchez-Beato M Orfao A Pintado B Flores T Sanchez-Martin M Jimenez R Piris MA Sanchez-Garcia I Cancer induction by restriction of oncogene expression to the stem cell compartment Embo J 2009 28 1 8 20 10.1038/emboj.2008.253 19037256 PMC2600654

[B9] Hahn WC Counter CM Lundberg AS Beijersbergen RL Brooks MW Weinberg RA Creation of human tumour cells with defined genetic elements Nature 1999 400 6743 464 468 10.1038/22780 10440377

[B10] Kho AT Zhao Q Cai Z Butte AJ Kim JY Pomeroy SL Rowitch DH Kohane IS Conserved mechanisms across development and tumorigenesis revealed by a mouse development perspective of human cancers Genes Dev 2004 18 6 629 640 10.1101/gad.1182504 15075291 PMC387239

[B11] Hu M Shivdasani RA Overlapping gene expression in fetal mouse intestine development and human colorectal cancer Cancer Res 2005 65 19 8715 8722 10.1158/0008-5472.CAN-05-0700 16204040

[B12] Kaiser S Park YK Franklin JL Halberg RB Yu M Jessen WJ Freudenberg J Chen X Haigis K Jegga AG Kong S Sakthivel B Xu H Reichling T Azhar M Boivin GP Roberts RB Bissahoyo AC Gonzales F Bloom GC Eschrich S Carter SL Aronow JE Kleimeyer J Kleimeyer M Ramaswamy V Settle SH Boone B Levy S Graff JM Transcriptional recapitulation and subversion of embryonic colon development by mouse colon tumor models and human colon cancer Genome Biol 2007 8 7 R131 10.1186/gb-2007-8-7-r131 17615082 PMC2323222

[B13] Ben-Porath I Thomson MW Carey VJ Ge R Bell GW Regev A Weinberg RA An embryonic stem cell-like gene expression signature in poorly differentiated aggressive human tumors Nat Genet 2008 40 5 499 507 10.1038/ng.127 18443585 PMC2912221

[B14] Mani SA Guo W Liao MJ Eaton EN Ayyanan A Zhou AY Brooks M Reinhard F Zhang CC Shipitsin M Campbell LL Polyak K Brisken C Yang J Weinberg RA The epithelial-mesenchymal transition generates cells with properties of stem cells Cell 2008 133 4 704 715 10.1016/j.cell.2008.03.027 18485877 PMC2728032

[B15] Morel AP Lievre M Thomas C Hinkal G Ansieau S Puisieux A Generation of breast cancer stem cells through epithelial-mesenchymal transition PLoS ONE 2008 3 8 e2888 10.1371/journal.pone.0002888 18682804 PMC2492808

[B16] Liu FS Molecular carcinogenesis of endometrial cancer Taiwan J Obstet Gynecol 2007 46 1 26 32 10.1016/S1028-4559(08)60102-3 17389185

[B17] Sorosky JI Endometrial cancer Obstet Gynecol 2008 111 2 Pt 1 436 447 18238985 10.1097/AOG.0b013e318162f690

[B18] Barker N Ridgway RA van Es JH Wetering M van de Begthel H Born M van den Danenberg E Clarke AR Sansom OJ Clevers H Crypt stem cells as the cells-of-origin of intestinal cancer Nature 2009 457 7229 608 611 10.1038/nature07602 19092804

[B19] Zhu L Gibson P Currle DS Tong Y Richardson RJ Bayazitov IT Poppleton H Zakharenko S Ellison DW Gilbertson RJ Prominin 1 marks intestinal stem cells that are susceptible to neoplastic transformation Nature 2009 457 7229 603 607 10.1038/nature07589 19092805 PMC2633030

[B20] Flier LG van der Haegebarth A Stange DE Wetering M van de Clevers H OLFM4 is a robust marker for stem cells in human intestine and marks a subset of colorectal cancer cells Gastroenterology 2009 137 1 15 17 10.1053/j.gastro.2009.05.035 19450592

[B21] Wang HW Trotter MW Lagos D Bourboulia D Henderson S Makinen T Elliman S Flanagan AM Alitalo K Boshoff C Kaposi sarcoma herpesvirus-induced cellular reprogramming contributes to the lymphatic endothelial gene expression in Kaposi sarcoma Nat Genet 2004 36 7 687 693 10.1038/ng1384 15220918

[B22] Huang TS Hsieh JY Wu YH Jen CH Tsuang YH Chiou SH Partanen J Anderson H Jaatinen T Yu YH Wang HW Functional network reconstruction reveals somatic stemness genetic maps and dedifferentiation-like transcriptome reprogramming induced by GATA2 Stem Cells 2008 26 5 1186 1201 10.1634/stemcells.2007-0821 18308945

[B23] Golub TR Slonim DK Tamayo P Huard C Gaasenbeek M Mesirov JP Coller H Loh ML Downing JR Caligiuri MA Bloomfield CD Lander ES Molecular classification of cancer: class discovery and class prediction by gene expression monitoring Science 1999 286 5439 531 537 10.1126/science.286.5439.531 10521349

[B24] Ramaswamy S Ross KN Lander ES Golub TR A molecular signature of metastasis in primary solid tumors Nat Genet 2003 33 1 49 54 10.1038/ng1060 12469122

[B25] Jen CH Yang TP Tung CY Su SH Lin CH Hsu MT Wang HW Signature Evaluation Tool (SET): a Java-based tool to evaluate and visualize the sample discrimination abilities of gene expression signatures BMC Bioinformatics 2008 9 1 58 10.1186/1471-2105-9-58 18221568 PMC2248562

[B26] Shabo I Stal O Olsson H Dore S Svanvik J Breast cancer expression of CD163, a macrophage scavenger receptor, is related to early distant recurrence and reduced patient survival Int J Cancer 2008 123 4 780 786 10.1002/ijc.23527 18506688

[B27] Komohara Y Ohnishi K Kuratsu J Takeya M Possible involvement of the M2 anti-inflammatory macrophage phenotype in growth of human gliomas J Pathol 2008 216 1 15 24 10.1002/path.2370 18553315

[B28] Uharcek P Prognostic factors in endometrial carcinoma J Obstet Gynaecol Res 2008 34 5 776 783 10.1111/j.1447-0756.2008.00796.x 18958927

[B29] Grushko TA Filiaci VL Mundt AJ Ridderstrale K Olopade OI Fleming GF An exploratory analysis of HER-2 amplification and overexpression in advanced endometrial carcinoma: a Gynecologic Oncology Group study Gynecol Oncol 2008 108 1 3 9 10.1016/j.ygyno.2007.09.007 17945336 PMC2699629

[B30] Strojan P Budihna M Smid L Svetic B Vrhovec I Kos J Skrk J Prognostic significance of cysteine proteinases cathepsins B and L and their endogenous inhibitors stefins A and B in patients with squamous cell carcinoma of the head and neck Clin Cancer Res 2000 6 3 1052 1062 10741734

[B31] Byers RJ Sakhinia E Joseph P Glennie C Hoyland JA Menasce LP Radford JA Illidge T Clinical quantitation of immune signature in follicular lymphoma by RT-PCR-based gene expression profiling Blood 2008 111 9 4764 4770 10.1182/blood-2007-10-115915 18174380

[B32] Salvesen HB Akslen LA Molecular pathogenesis and prognostic factors in endometrial carcinoma APMIS 2002 110 10 673 689 10.1034/j.1600-0463.2002.1101001.x 12583434

[B33] An C Choi IS Yao JC Worah S Xie K Mansfield PF Ajani JA Rashid A Hamilton SR Wu TT Prognostic significance of CpG island methylator phenotype and microsatellite instability in gastric carcinoma Clin Cancer Res 2005 11 2 Pt 1 656 663 15701853

[B34] Toyota M Kopecky KJ Toyota MO Jair KW Willman CL Issa JP Methylation profiling in acute myeloid leukemia Blood 2001 97 9 2823 2829 10.1182/blood.V97.9.2823 11313277

[B35] Bieche I Lerebours F Tozlu S Espie M Marty M Lidereau R Molecular profiling of inflammatory breast cancer: identification of a poor-prognosis gene expression signature Clin Cancer Res 2004 10 20 6789 6795 10.1158/1078-0432.CCR-04-0306 15501955

[B36] Skibbens RV Cell biology of cancer: BRCA1 and sister chromatid pairing reactions? Cell Cycle 2008 7 4 449 452 18235242 10.4161/cc.7.4.5435

[B37] Juric D Sale S Hromas RA Yu R Wang Y Duran GE Tibshirani R Einhorn LH Sikic BI Gene expression profiling differentiates germ cell tumors from other cancers and defines subtype-specific signatures Proc Natl Acad Sci USA 2005 102 49 17763 17768 10.1073/pnas.0509082102 16306258 PMC1308932

[B38] Kho DH Bae JA Lee JH Cho HJ Cho SH Seo YW Ahn KY Chung IJ Kim KK KITENIN recruits Dishevelled/PKC delta to form a functional complex and controls the migration and invasiveness of colorectal cancer cells Gut 2009 58 4 509 519 10.1136/gut.2008.150938 18653728

[B39] Birnie R Bryce SD Roome C Dussupt V Droop A Lang SH Berry PA Hyde CF Lewis JL Stower MJ Maitland NJ Collins AT Gene expression profiling of human prostate cancer stem cells reveals a pro-inflammatory phenotype and the importance of extracellular matrix interactions Genome Biol 2008 9 5 R83 10.1186/gb-2008-9-5-r83 18492237 PMC2441469

[B40] Arribas J Esselens C ADAM17 as a therapeutic target in multiple diseases Curr Pharm Des 2009 15 20 2319 2335 10.2174/138161209788682398 19601834

[B41] Yamazaki K Takamura M Masugi Y Mori T Du W Hibi T Hiraoka N Ohta T Ohki M Hirohashi S Sakamoto M Adenylate cyclase-associated protein 1 overexpressed in pancreatic cancers is involved in cancer cell motility Lab Invest 2009 89 4 425 432 10.1038/labinvest.2009.5 19188911

[B42] Thompson CC Ashcroft FJ Patel S Saraga G Vimalachandran D Prime W Campbell F Dodson A Jenkins RE Lemoine NR Crnogorac-Jurcevic T Yin HL Costello E Pancreatic cancer cells overexpress gelsolin family-capping proteins, which contribute to their cell motility Gut 2007 56 1 95 106 10.1136/gut.2005.083691 16847067 PMC1856675

[B43] Labauge P Denier C Bergametti F Tournier-Lasserve E Genetics of cavernous angiomas Lancet Neurol 2007 6 3 237 244 10.1016/S1474-4422(07)70053-4 17303530

[B44] Ma X Zhao H Shan J Long F Chen Y Zhang Y Han X Ma D PDCD10 interacts with Ste20-related kinase MST4 to promote cell growth and transformation via modulation of the ERK pathway Mol Biol Cell 2007 18 6 1965 1978 10.1091/mbc.E06-07-0608 17360971 PMC1877091

[B45] Lu Y Lemon W Liu PY Yi Y Morrison C Yang P Sun Z Szoke J Gerald WL Watson M Govindan R You M A gene expression signature predicts survival of patients with stage I non-small cell lung cancer PLoS Med 2006 3 12 e467 10.1371/journal.pmed.0030467 17194181 PMC1716187

[B46] Chiu SY Asai N Costantini F Hsu W SUMO-specific protease 2 is essential for modulating p53-Mdm2 in development of trophoblast stem cell niches and lineages PLoS Biol 2008 6 12 e310 10.1371/journal.pbio.0060310 19090619 PMC2602722

[B47] Hoffman AE Zheng T Ba Y Zhu Y The circadian gene NPAS2, a putative tumor suppressor, is involved in DNA damage response Mol Cancer Res 2008 6 9 1461 1468 10.1158/1541-7786.MCR-07-2094 18819933 PMC2572675

[B48] Deshpande AM Akunowicz JD Reveles XT Patel BB Saria EA Gorlick RG Naylor SL Leach RJ Hansen MF PHC3, a component of the hPRC-H complex, associates with E2F6 during G0 and is lost in osteosarcoma tumors Oncogene 2007 26 12 1714 1722 10.1038/sj.onc.1209988 17001316 PMC2691996

[B49] Chen HY Yu SL Chen CH Chang GC Chen CY Yuan A Cheng CL Wang CH Terng HJ Kao SF Chan WK Li HN Liu CC Singh S Chen WJ Chen JJ Yang PC A five-gene signature and clinical outcome in non-small-cell lung cancer N Engl J Med 2007 356 1 11 20 10.1056/NEJMoa060096 17202451

[B50] Yu SL Chen HY Chang GC Chen CY Chen HW Singh S Cheng CL Yu CJ Lee YC Chen HS Su TJ Chiang CC Li HN Hong QS Su HY Chen CC Chen WJ Liu CC Chan WK Li KC Chen JJ Yang PC MicroRNA signature predicts survival and relapse in lung cancer Cancer Cell 2008 13 1 48 57 10.1016/j.ccr.2007.12.008 18167339

[B51] Weigelt B Glas AM Wessels LF Witteveen AT Peterse JL van't Veer LJ Gene expression profiles of primary breast tumors maintained in distant metastases Proc Natl Acad Sci USA 2003 100 26 15901 15905 10.1073/pnas.2634067100 14665696 PMC307665

[B52] Podsypanina K Du YC Jechlinger M Beverly LJ Hambardzumyan D Varmus H Seeding and propagation of untransformed mouse mammary cells in the lung Science 2008 321 5897 1841 1844 10.1126/science.1161621 18755941 PMC2694414

[B53] Husemann Y Geigl JB Schubert F Musiani P Meyer M Burghart E Forni G Eils R Fehm T Riethmuller G Klein CA Systemic spread is an early step in breast cancer Cancer Cell 2008 13 1 58 68 10.1016/j.ccr.2007.12.003 18167340

[B54] Chang SJ Huang TS Wang KL Wang TY Yang YC Chang MD Wu YH Wang HW Genetic network analysis of human CD34+ hematopoietic stem/precursor cells Taiwanese Journal of Obstetrics & Gynecology 2008 47 4 422 430 10.1016/S1028-4559(09)60010-3 19126509

[B55] Yang TP Chang TY Lin CH Hsu MT Wang HW ArrayFusion: a web application for multi-dimensional analysis of CGH, SNP and microarray data Bioinformatics 2006 22 21 2697 2698 10.1093/bioinformatics/btl457 16935928

[B56] Harris MA Clark J Ireland A Lomax J Ashburner M Foulger R Eilbeck K Lewis S Marshall B Mungall C Richter J Rubin GM Blake JA Bult C Dolan M Drabkin H Eppig JT Hill DP Ni L Ringwald M Balakrishnan R Cherry JM Christie KR Costanzo MC Dwight SS Engel S Fisk DG Hirschman JE Hong EL Nash RS The Gene Ontology (GO) database and informatics resource Nucleic Acids Res 2004 32 Database D258 261 14681407 10.1093/nar/gkh036 PMC308770

[B57] Dennis G Jr Sherman BT Hosack DA Yang J Gao W Lane HC Lempicki RA DAVID: Database for Annotation, Visualization, and Integrated Discovery Genome Biol 2003 4 5 P3 10.1186/gb-2003-4-5-p3 12734009 PMC193660

