# Supplementary figures and images for: Increased epithelial stem cell traits in advanced endometrial endometrioid carcinoma
Source: BMC Genomics. 2009 Dec 16;10:613. doi: 10.1186/1471-2164-10-613 (PMC2810306; doi:10.1186/1471-2164-10-613)

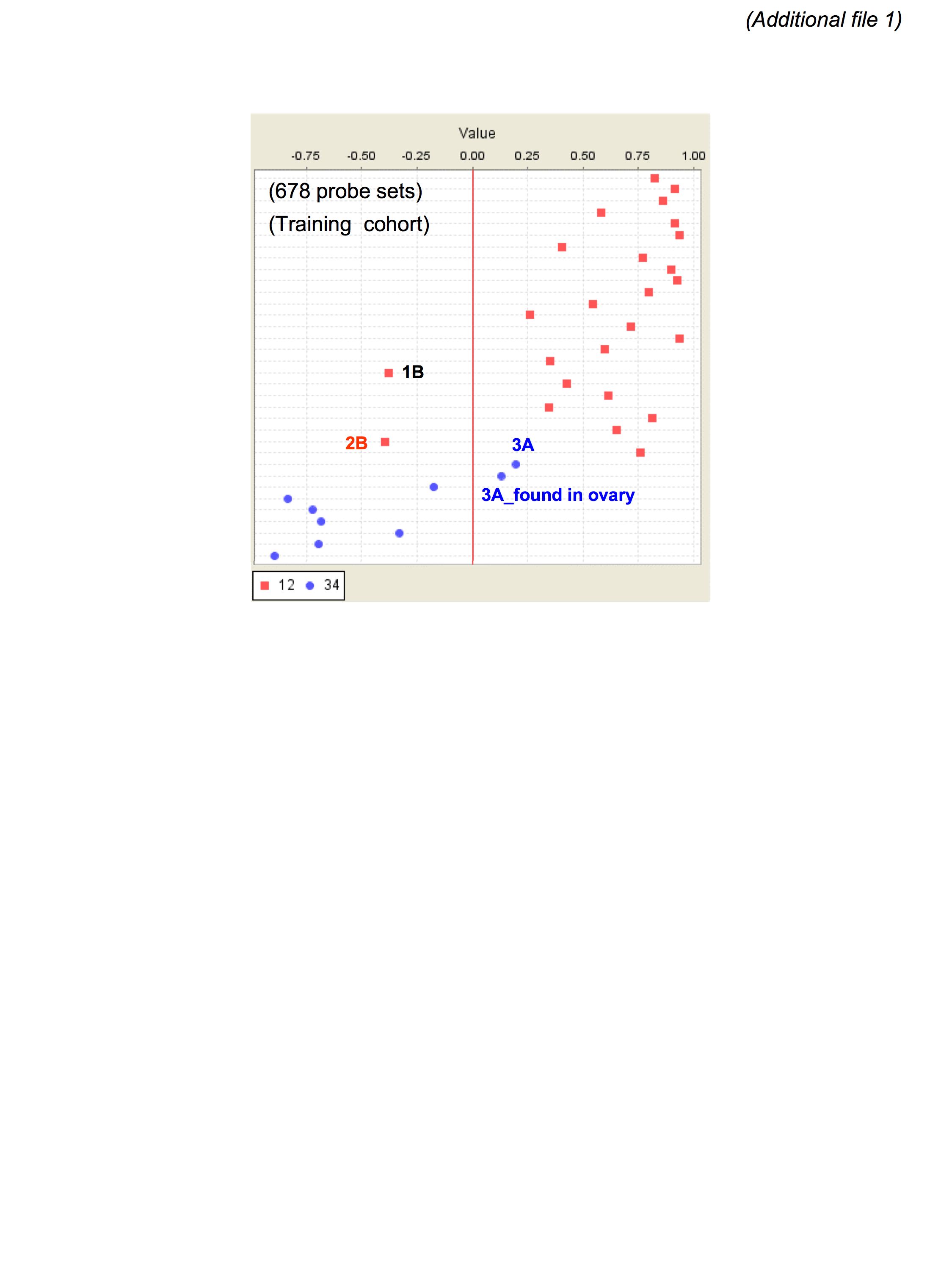

Supplement: Additional file 1 — The discrimination ability of the 678 probe sets. Prediction power of the 678 probe sets differentiating early and late stage samples, as well as discriminating normal endometrium and tumor tissues. [file 1471-2164-10-613-S1.JPEG]
